# Relevance of the MHC region for breast cancer susceptibility in Asians

**DOI:** 10.1007/s12282-022-01366-w

**Published:** 2022-05-11

**Authors:** Peh Joo Ho, Alexis Jiaying Khng, Benita Kiat-Tee Tan, Ern Yu Tan, Su-Ming Tan, Veronique Kiak Mien Tan, Geok Hoon Lim, Kristan J. Aronson, Tsun L. Chan, Ji-Yeob Choi, Joe Dennis, Weang-Kee Ho, Ming-Feng Hou, Hidemi Ito, Motoki Iwasaki, Esther M. John, Daehee Kang, Sung-Won Kim, Allison W. Kurian, Ava Kwong, Artitaya Lophatananon, Keitaro Matsuo, Nur Aishah Mohd-Taib, Kenneth Muir, Rachel A. Murphy, Sue K. Park, Chen-Yang Shen, Xiao-Ou Shu, Soo Hwang Teo, Qin Wang, Taiki Yamaji, Wei Zheng, Manjeet K. Bolla, Alison M. Dunning, Douglas F. Easton, Paul D. P. Pharoah, Mikael Hartman, Jingmei Li

**Affiliations:** 1 Women’s Health and Genetics, Genome Institute of Singapore, 60 Biopolis Street, Genome, #02-01, Singapore, 138672 Singapore; 2 Saw Swee Hock School of Public Health, National University of Singapore and National University Health System, Singapore, 119077 Singapore; 3 Department of Surgery, National University Health System, Singapore, 119228 Singapore; 4 Department of Breast Surgery, Singapore General Hospital, Singapore, Singapore; 5 Division of Surgery and Surgical Oncology, National Cancer Centre Singapore, Singapore, Singapore; 6 Department of General Surgery, Sengkang General Hospital, Singapore, Singapore; 7 Department of General Surgery, Tan Tock Seng Hospital, Singapore, 308433 Singapore; 8 Lee Kong Chian School of Medicine, Singapore, Singapore; 9 Institute of Molecular and Cell Biology, Singapore, Singapore; 10 Division of Breast Surgery, Changi General Hospital, Singapore, Singapore; 11 KK Breast Department, KK Women’s and Children’s Hospital, Singapore, 229899 Singapore; 12 Department of Public Health Sciences, and Cancer Research Institute, Queen’s University, Kingston, ON K7L 3N6 Canada; 13 Hong Kong Hereditary Breast Cancer Family Registry, Hong Kong, Hong Kong; 14 Department of Molecular Pathology, Hong Kong Sanatorium and Hospital, Hong Kong, Hong Kong; 15 Department of Biomedical Sciences, Seoul National University Graduate School, Seoul, 03080 Korea; 16 Cancer Research Institute, Seoul National University, Seoul, 03080 Korea; 17 Institute of Health Policy and Management, Seoul National University Medical Research Center, Seoul, 03080 Korea; 18 Department of Public Health and Primary Care, Centre for Cancer Genetic Epidemiology, University of Cambridge, Cambridge, CB1 8RN UK; 19 Department of Mathematical Sciences, Faculty of Science and Engineering, University of Nottingham Malaysia Campus, 43500 Semenyih, Selangor Malaysia; 20 Breast Cancer Research Programme, Cancer Research Malaysia, 47500 Subang Jaya, Selangor Malaysia; 21 Department of Surgery, Kaohsiung Municipal Hsiao-Kang Hospital, Kao-hsiung, 812 Taiwan; 22 Division of Cancer Information and Control, Aichi Cancer Center Research Institute, Nagoya, 464-8681 Japan; 23 Division of Cancer Epidemiology, Nagoya University Graduate School of Medicine, Nagoya, 466-8550 Japan; 24 Division of Epidemiology, Center for Public Health Sciences, National Cancer Center, Tokyo, 104-0045 Japan; 25 Department of Epidemiology and Population Health, Stanford University School of Medicine, Stanford, CA 94305 USA; 26 Department of Medicine, Division of Oncology, Stanford Cancer Institute, Stanford University School of Medicine, Stanford, CA 94304 USA; 27 Department of Preventive Medicine, Seoul National University College of Medicine, Seoul, 03080 Korea; 28 Department of Surgery, Daerim Saint Mary’s Hospital, Seoul, 07442 Korea; 29 Department of Surgery, The University of Hong Kong, Hong Kong, Hong Kong; 30 Department of Surgery and Cancer Genetics Center, Hong Kong Sanatorium and Hospital, Hong Kong, Hong Kong; 31 Division of Population Health, Health Services Research and Primary Care, School of Health Sciences, Faculty of Biology, Medicine and Health, The University of Manchester, Manchester, M13 9PL UK; 32 Division of Cancer Epidemiology and Prevention, Aichi Cancer Center Research Institute, Nagoya, 464-8681 Japan; 33 Department of Surgery, Faculty of Medicine, University of Malaya, 50603 Kuala Lumpur, Malaysia; 34 School of Population and Public Health, University of British Columbia, Vancouver, BC V6T 1Z4 Canada; 35 Cancer Control Research, BC Cancer, Vancouver, BC V5Z 1L3 Canada; 36 Integrated Major in Innovative Medical Science, Seoul National University College of Medicine, Seoul, 03080 South Korea; 37 Institute of Biomedical Sciences, Academia Sinica, Taipei, 115 Taiwan; 38 School of Public Health, China Medical University, Taichung, Taiwan; 39 Division of Epidemiology, Department of Medicine, Vanderbilt Epidemiology Center, Vanderbilt-Ingram Cancer Center, Vanderbilt University School of Medicine, Nashville, TN 37232 USA; 40 Department of Oncology, Centre for Cancer Genetic Epidemiology, University of Cambridge, Cambridge, CB1 8RN UK

**Keywords:** HLA, Breast cancer risk, Breast cancer subtypes

## Abstract

**Background:**

Human leukocyte antigen (HLA) genes play critical roles in immune surveillance, an important defence against tumors. Imputing HLA genotypes from existing single-nucleotide polymorphism datasets is low-cost and efficient. We investigate the relevance of the major histocompatibility complex region in breast cancer susceptibility, using imputed class I and II HLA alleles, in 25,484 women of Asian ancestry.

**Methods:**

A total of 12,901 breast cancer cases and 12,583 controls from 12 case–control studies were included in our pooled analysis. HLA imputation was performed using SNP2HLA on 10,886 quality-controlled variants within the 15–55 Mb region on chromosome 6. HLA alleles (*n* = 175) with info scores greater than 0.8 and frequencies greater than 0.01 were included (resolution at two-digit level: 71; four-digit level: 104). We studied the associations between HLA alleles and breast cancer risk using logistic regression, adjusting for population structure and age. Associations between HLA alleles and the risk of subtypes of breast cancer (ER-positive, ER-negative, HER2-positive, HER2-negative, early-stage, and late-stage) were examined.

**Results:**

We did not observe associations between any HLA allele and breast cancer risk at *P* < 5e−8; the smallest p value was observed for HLA-C*12:03 (OR = 1.29, *P* = 1.08e−3). Ninety-five percent of the effect sizes (OR) observed were between 0.90 and 1.23. Similar results were observed when different subtypes of breast cancer were studied (95% of ORs were between 0.85 and 1.18).

**Conclusions:**

No imputed HLA allele was associated with breast cancer risk in our large Asian study. Direct measurement of HLA gene expressions may be required to further explore the associations between HLA genes and breast cancer risk.

**Supplementary Information:**

The online version contains supplementary material available at 10.1007/s12282-022-01366-w.

## Introduction

The major histocompatibility complex (MHC) region in the human genome is gene dense, highly polymorphic, and known as a hotspot for disease associations (mainly autoimmune and infectious). Mapping to the short arm of chromosome 6, the MHC region includes human leukocyte antigen (HLA) genes and plays various critical roles in regulating immune response. One such function is immune surveillance, which is an important defense against tumors [1].

Many studies have examined HLA genotypes in the MHC region in relation to breast cancer susceptibility [2–4]. However, low statistical power due to the limited sample sizes in these studies has made it difficult to obtain consistent and convincing results. Nonetheless, in a large-scale GWAS study comprising 62,533 breast cancer cases and 60,976 controls of European ancestry, a variant within the MHC region (rs9257408, chr6: 28,926,220) was found to be significantly associated with the risk of breast malignancy [5]. Pathway analyses of a larger, follow-up GWAS study comprising over 200,000 individuals revealed that immune-related processes may underlie some of the observed associations with breast cancer susceptibility [6].

Traditional GWAS analyses do not go beyond the interrogation of individual single-nucleotide polymorphisms (SNPs). The MHC region is unique in that beyond SNPs, there is an additional layer of functional polymorphisms in the form of HLA genotypes [7]. Transforming SNP-based associations into HLA-allele level associations may thus yield more information and biological meaning behind the associations [8].

Probe-based genotyping commonly used in GWAS is suboptimal for querying variation in the MHC region due to its extensive polymorphic nature [9]. However, with the recent development of new computational tools and large reference panels, HLA genotypes can be inferred from SNPs that are in close proximity to classical HLA loci. Compared to serological typing and direct sequencing approaches, imputation is a less accurate method of obtaining HLA genotype calls. However, determining HLA genotypes in silico using readily available SNP datasets has the benefits of being low-cost and efficient in both time and labor. We aim to investigate the relevance of the MHC region in breast cancer susceptibility, using imputed HLA alleles, in 25,484 women of Asian ancestry.

## Methods

### Study population

We studied 25,484 women from 12 case–control studies participating in the Breast Cancer Association Consortium (BCAC). These women were genetically Asian with known breast cancer status. Our study includes a total of 12,901 breast cancer patients and 12,583 controls (Supplementary Table 1 and Supplementary Fig. 1). This study was approved by the ethics board of the Agency for Science, Technology and Research (ASTAR IRB Ref: 2020-154). Informed consent was obtained by the individual studies which contributed to BCAC.

### Single nucleotide variants (SNPs)

DNA was genotyped using OncoArray (Illumina Infinium array) [6, 10]. A data request application was made to BCAC to obtain all variants within the 15–55 Mb region (build 37) on chromosome 6 (concept 686); of which 15,412 variants were received. A total of 10,886 variants remained after excluding 181 variants with missing genotype data and 4345 variants with minor allele frequency (MAF) below 0.01. The total genotyping rate was 0.999.

### Imputation of human leukocyte antigen (HLA) alleles

We imputed 273 HLA alleles (in dosage format; 94 classical 2-digit alleles and 179 classical 4-digit alleles.) using the SNP2HLA package (v1.0.3) with using a pre-built reference panel (available as part of the software) based on the data of pan-Asian subjects (including Han Chinese, Southeast Asian Malay, Tamil Indian ancestries, and Japanese) [11, 12]. Imputation was done in batches of 100 individuals. The info score for each individual was calculated from all individuals, based on the ratio of empirical and expected variance in dosage. The frequency of HLA alleles present ranges from 0.0005 to 0.489 (Supplementary Table 2). HLA alleles (*n* = 175) with info scores greater than 0.8 and frequency greater than 0.01 were selected for association analysis [9].

### Breast cancer risk factors

Information on breast cancer risk factors was collected at enrollment for nested case–control studies, or post breast cancer diagnosis for cases in case–control studies. Risk factors used in our study: number of first-degree family members with breast cancer (none, 1,  ≥ 2), body mass index (BMI), age at menarche (years), parity, menopausal status (post-menopausal, pre-/peri-menopausal), oral contraceptive use (≥ 4 months, < 4 months), and hormone replacement therapy use (HRT; ≥ 3 months, < 3 months). Women were considered post-menopausal if their date of last menstruation was > 12 months prior to the date of breast cancer diagnosis for cases or enrollment for controls. Ethnicity was self-reported with the exception of Korean and Thai, where they were coded based on the study’s country.

### Statistical analysis

Pooled analysis was performed as the mean and standard deviation of the HLA alleles were similar across studies (Supplementary Figs. 2 and 3). To study the associations of SNPs and HLA alleles with breast cancer status, logistic regression was used with adjustment for age (at recruitment for controls, and at diagnosis for cases) and the first 15 principal components (PC) (i.e. population structure). The PCs were based on 22 chromosomes. Further adjustment for breast cancer risk factors (study, family history, BMI, parity, age at menarche, menopausal status, contraceptive use, and hormone replacement therapy use) was done for the study of HLA alleles with breast cancer status. To compare our results with published literature, we did a systematic review of studies on HLA and breast cancer risk in Asian patients (Supplementary Methods).

Breast cancer tumor characteristics were obtained from medical records. The association analysis, using logistic regression, was repeated with all controls and subgroups of cases, (1) estrogen receptor (ER) positive, (2) ER-negative, (3) human epidermal growth factor receptor 2 (HER2) positive, (4) HER2-negative, (5) early-stage (stages 0 or I), and (6) late-stage (stage III or IV).

## Results

### Ethnicity distribution in our study

**Fig. 1:**
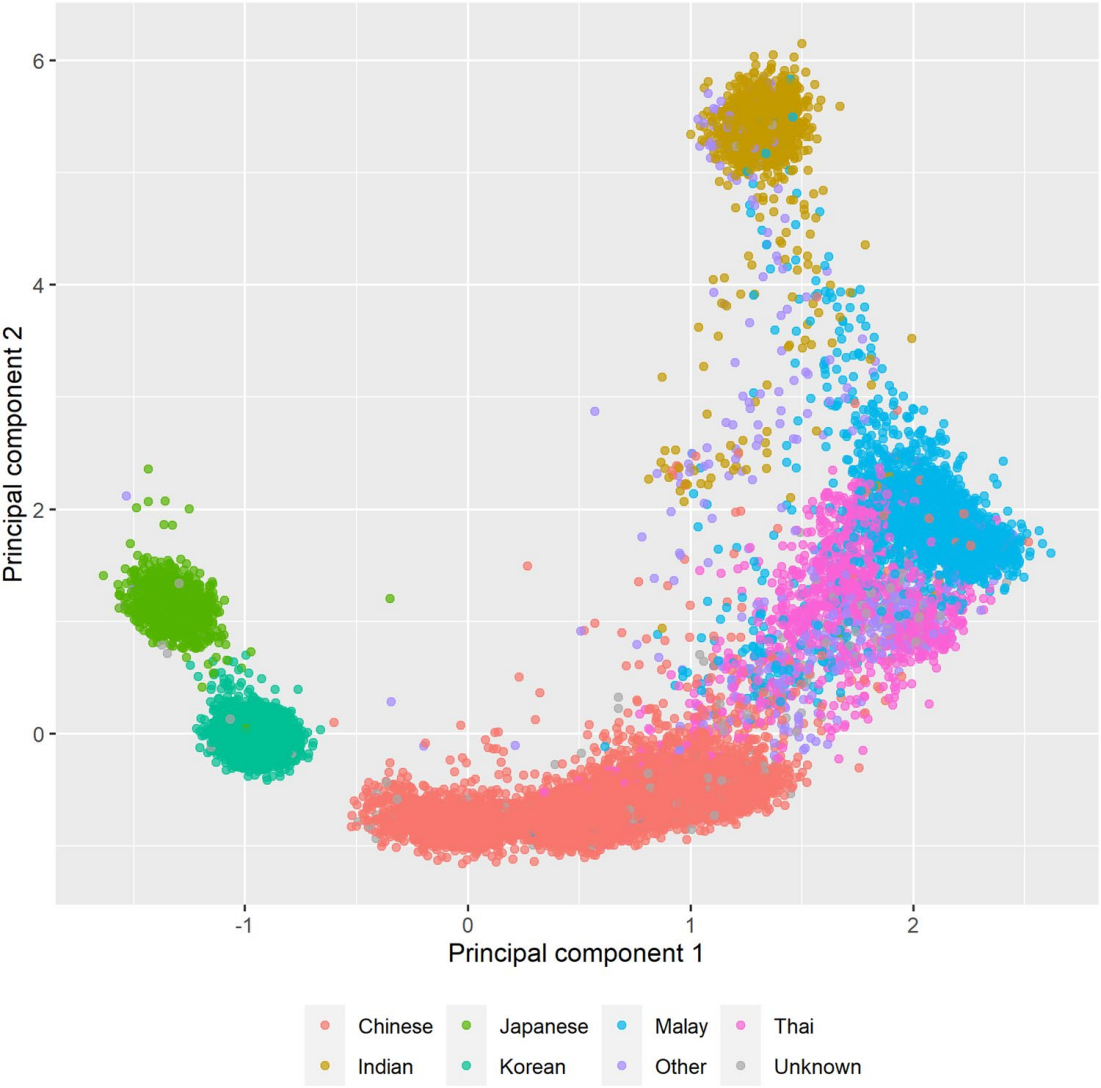
Principal components plot plotting principal component 1 and 2 colored by ethnicity

A PC analysis (PCA) plot of genotyping data shows the distinction between the six major ethnicities present in our study (Fig. 1). Over half of all women were Chinese (*n* = 14,048), and were from seven of the twelve studies (Table 1, Supplementary Table 3). The same PCA plot, but colored by study, showed that the spread seen in the Chinese is due to the country of recruitment (Supplementary Fig. 1). Indians were from multi-ethnic Singapore and Malaysia (Supplementary Table 3).

**Table 1:** Characteristics of Asian breast cancer cases and controls from 12 studies (*n* = 25,484)

	Controls	Cases
	*n* = 12,583	*n* = 12,901
Age, years (at recruitment for controls, at diagnosis for cases)	51 (44–58)	52 (45–60)
Unknown	234	34
Ethnicity		
Chinese	6,809 (54)	7239 (56)
Indian	1019 (8)	577 (4)
Japanese	657 (5)	661 (5)
Korean^a^	1772 (14)	2394 (19)
Malay	1335 (11)	1061 (8)
Thai^a^	642 (5)	448 (3)
Other	161 (1)	243 (2)
Unknown	188 (1)	278 (2)
Family history of breast cancer		
None	6753 (54)	8885 (69)
1	607 (5)	1,265 (10)
≥ 2	89 (1)	192 (1)
Unknown	5134 (41)	2559 (20)
Body mass index, kg/m^2^		
< 18.5	566 (4)	584 (5)
18.5–24.9	7226 (57)	5458 (42)
25.0–29.9	3292 (26)	2189 (17)
≥ 30	1140 (9)	705 (5)
Unknown	359 (3)	3965 (31)
Age at menarche, years		
≤ 12	3708 (29)	3567 (28)
13–14	5024 (40)	4320 (33)
> 14	3087 (25)	2356 (18)
Unknown	764 (6)	2658 (21)
Parity		
Nulliparous	1823 (14)	1940 (15)
1 child	1719 (14)	1926 (15)
2 children	4276 (34)	3434 (27)
≥ 3 children	4168 (33)	3320 (26)
Unknown	597 (5)	2281 (18)
Menopausal status		
Postmenopausal	5718 (45)	5603 (43)
Premenopausal or peri-menopausal	6028 (48)	3989 (31)
Unknown	837 (7)	3309 (26)
Oral contraceptive		
Never	4227 (34)	4828 (37)
Ever	1125 (9)	1841 (14)
Unknown	7231 (57)	6232 (48)
Hormone replacement therapy		
Never	5088 (40)	8199 (64)
Ever	682 (5)	749 (6)
Unknown	6813 (54)	3953 (31)

^a^We re-coded Korean and Thai from other ethnicities based on the country (Korea and Thailand, respectively) the study was from

### Characteristics of cases and controls

The median age at breast cancer diagnosis for cases (*n* = 12,901) was 52 years (interquartile range [IQR]: 45–60) and the median age at enrollment for controls (*n* = 12,583) was 51 years (IQR: 44–58) (Table 1). The majority of all women were of normal BMI (18.5–24.9 kg/m^2^, 42% in cases, and 57% in controls). The majority of cases (68%) and controls (81%) had at least one child. Forty-three percent of cases were post-menopausal at the time of diagnosis, a similar proportion of post-menopausal controls (45%) were recruited. Older age at menarche was observed in some studies (Asia Cancer Program [ACP], Korean Hereditary Breast Cancer Study [KOHBRA], Shanghai Breast Cancer Genetic Study [SBCGS], Seoul Breast Cancer Study [SEBCS], and Taiwanese Breast Cancer Study [TWBCS]) (Supplementary Table 4). The majority of breast cancer cases were ER-positive (*n* = 8558, 66%), HER2-negative (*n* = 3777, 45%), and stage II (*n* = 4597, 36%) (Supplementary Table 1).

### Associations between SNPs and breast cancer risk

**Fig. 2:**
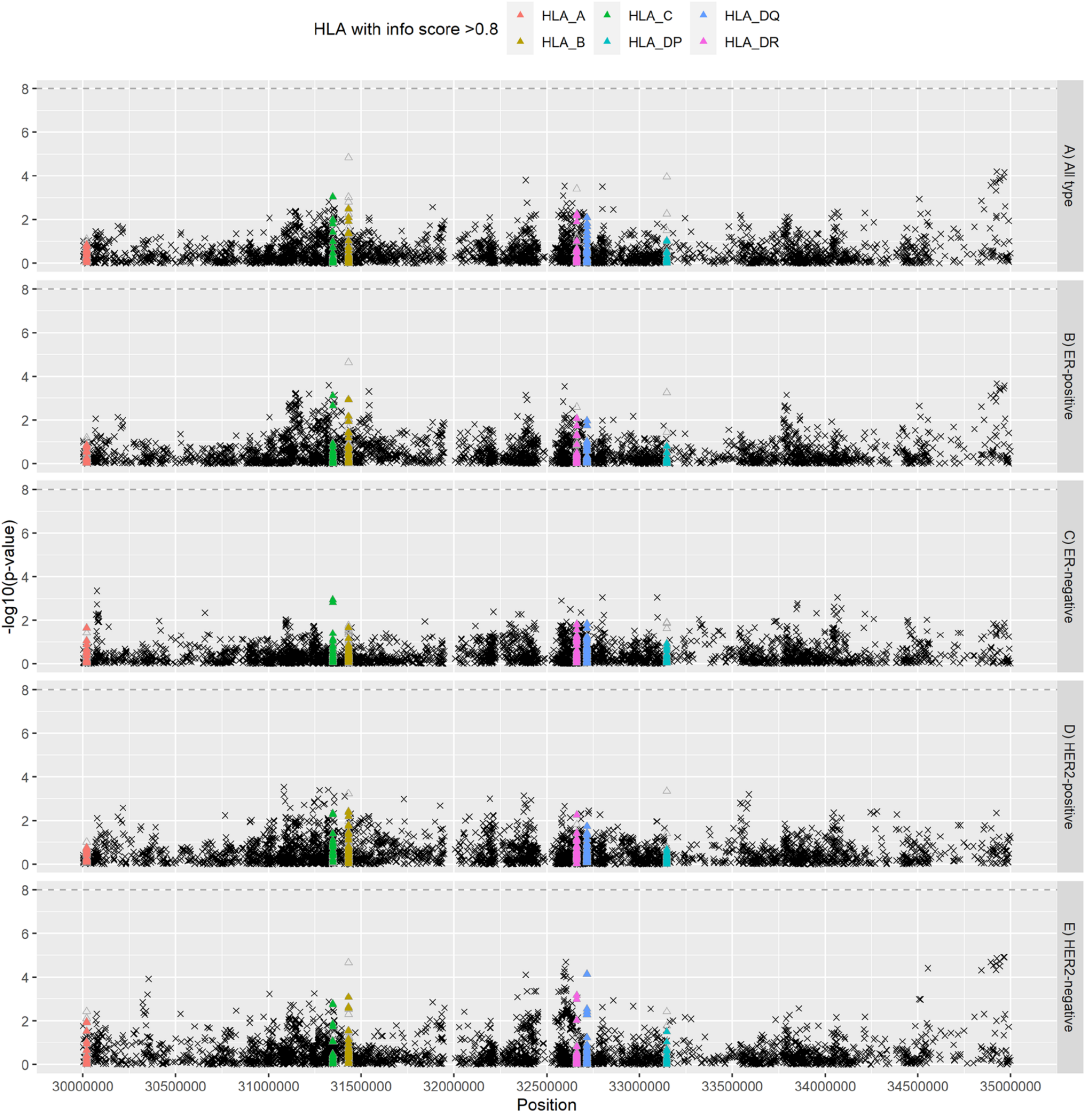
Manhattan plot of the associations between imputed human leukocyte antigen alleles (HLA, *n* = 273; triangles; as predicted by SNP2HLA), single nucleotide polymorphisms (SNP) on chromosome 6 (30–35 Mb, build 37) and the risk of developing breast cancer as compared to controls (*n* = 12,583)—**a** all types (*n* = 12,901), **b** estrogen receptor (ER)-positive (*n* = 8558), **c** ER-negative (*n* = 3722), **d** HER2-positive (*n* = 3722), and **e** HER2-negative (*n* = 5848). Filled triangles denote associations with HLA (*n* = 175) with high (≥ 0.8) info score and frequency > 0.01; unfilled triangles denote HLA class. Logistic regression models were used and adjusted for the first 15 principal components for population stratification and age (at recruitment for controls; at diagnosis for cases). Info score: info score from HLA prediction by SNP2HLA

**Fig. 3:**
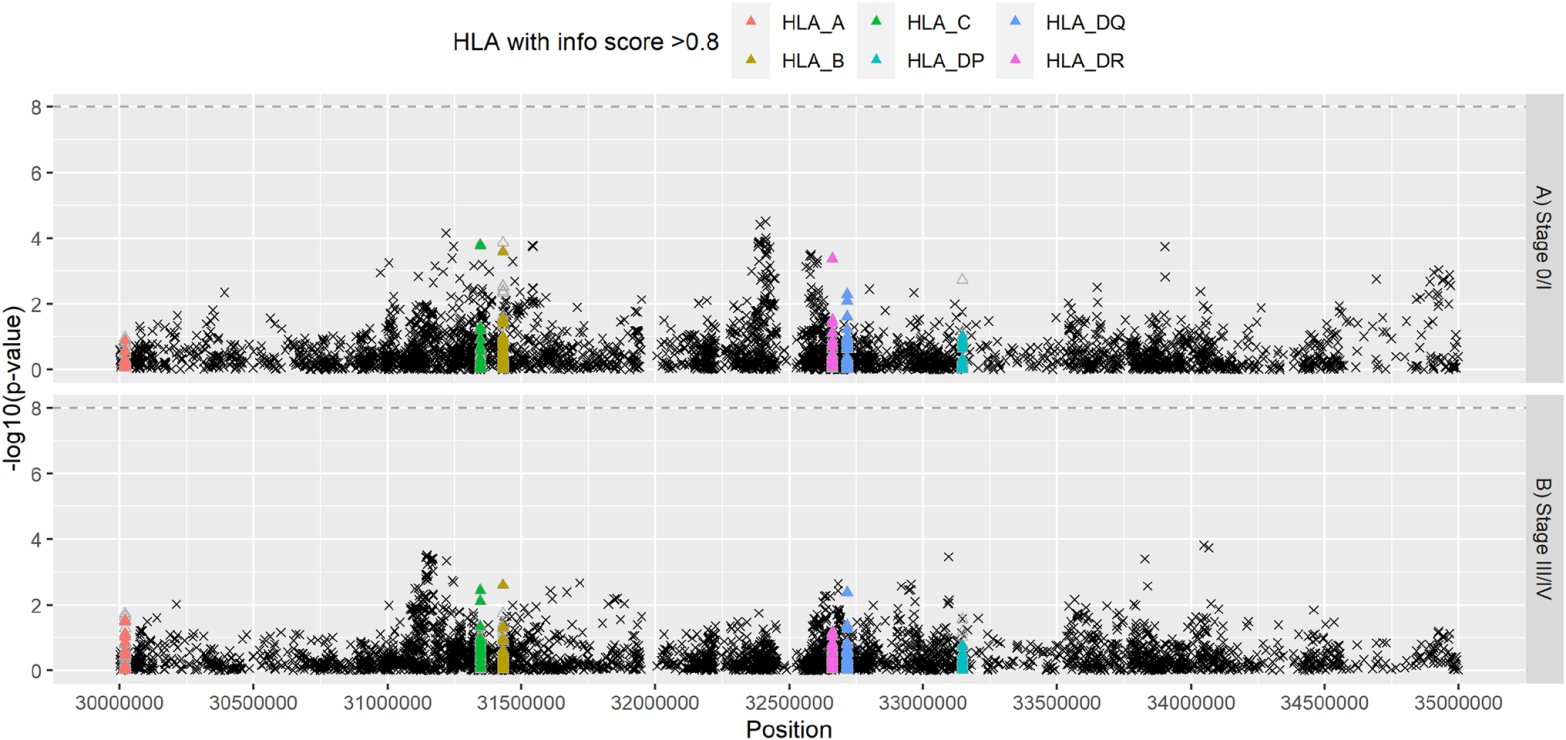
Manhattan plot of the associations between imputed human leukocyte antigen alleles (HLA, *n* = 273; triangles; as predicted by SNP2HLA), single nucleotide polymorphisms (SNP) on chromosome 6 (30–35 Mb, build 37) and the risk of developing breast cancer as compared to controls (*n* = 12,583)—**a** stage 0 or I (*n* = 3896), and **b** stage III or IV (*n* = 1972). Filled triangles denote associations with HLA (*n* = 175) with high (≥ 0.8) info score and frequency > 0.01; unfilled triangles denote HLA class. Logistic regression models were used and adjusted for the first 15 principle components for population stratification and age (at recruitment for controls; at diagnosis for cases). Info score: info score from HLA prediction by SNP2HLA

None of the associations between the 10,886 SNPs chromosome 6 (15–55 Mb) and breast cancer risk reached genome-wide significance (*P* < 5e−8) (Fig. 2A). The smallest p-value observed was for SNP rs12663096 (odds ratio [OR] = 0.92, *P* = 5.01e−05). Effect sizes (odds ratios, OR) observed ranged from 0.77 to 1.25, where 95% of the effect sizes observed were between 0.92 and 1.07. Similar non-significant results were observed when subgroups of breast cancer were studied, with the smallest *p*-value observed for rs9784889 and HER2-negative breast cancer (OR = 0.889, *P* = 1.19e−5) (Figs. 2B–E and Fig. 3).

### Associations between HLA alleles and breast cancer risk

Of the 273 imputed HLA alleles, 175 had info scores greater than 0.8 and frequencies greater than 0.01; seventy-one were classic 2-digits HLA alleles and 104 were classic 4-digits HLA alleles (Supplementary Table 5). Heatmap of the 71 classic 2-digits HLA alleles by study participants did not show obvious clustering of cases and controls (Supplementary Fig. 4).

Of the 175 HLA alleles, the association between HLA-C*12:03 and breast cancer risk attained the smallest p-value (OR = 1.29, *P* = 1.08e-3) (Fig. 2A). Effect sizes (OR) observed ranged from 0.87 to 1.29, where 95% of the effect sizes (OR) observed were between 0.90 and 1.23 (Supplementary Table 5). No appreciable change was observed after adjustments for study and breast cancer risk factors (age, body mass index number of first-degree family members with breast cancer, number of children, age at menarche, menopausal status, oral contraceptive use, and hormone replacement therapy use) (Supplementary Fig. 5A and Supplementary Table 56).

Three of 14 articles identified in the literature reported associations between classic HLA class I or class II alleles and breast cancer risk (Supplementary Methods). All studies used a case–control design and HLA ascertained from blood samples. Only Leong et al. reported significant associations between HLA-A*31 and breast cancer risk (Fisher’s exact test *P* = 0.020) (Table 2). Two other HLA alleles (HLA-A*26 and HLA-A*36) were reported to be correlated with metastasis (Table 2). We did not observe the same associations in our study; the associations between HLA-A*31and breast cancer risk (OR = 1.05, *P* = 0.324) and between HLA-A*26 and late-stage breast cancer (OR = 1.11, *P* = 0.260) were not significant. HLA-A*36 alleles were not imputed.

**Table 2:** Findings from the three articles that were identified from our systematic review (Supplementary Methods: a systematic review of studies on HLA in Asian breast cancer patients)

First author (year of publication)	Country	Sample size	HLA measurement	Results from literature	Our results^a^
Leong PP (2011)	Malaysia	59 cases; 77 controls	HLA-A Sequence-Specific primers PCR	HLA-A*31 (cases vs controls; Fisher's exact test, *P* = 0.020)	HLA-A*31 and breast cancer risk (Odds ratio [OR] = 1.05, *P* = 0.324)
				HLA-A*26 (Metastasis; Spearman's rank test, *r* = − 0.430, *P* = 0.001)	HLA-A*26 and breast cancer risk (OR = 0.96, *P* = 0.454)
				HLA-A*36 (Metastasis; Spearman's rank test, *r* = − 0.430, *P* = 0.001)	HLA-A*26 and late-stage breast cancer risk (OR = 1.11, *P* = 0.260)
					HLA-A*36 was not imputed
Yang XX (2011)	China	216 cases; 216 controls	16 variants in HLA class II region were genotyped	HLA class II variants studied were not associated with breast cancer risk	Associations for class II variants did not reach genome-wide significance (*P* < 5e−8)
Chen PC (2007)	Taiwan	101 cases; 115 controls	Genotyping was performed by Sequence-Specific primers PCR	No significant differences in phenotype frequencies of HLA-DQA1 and -DQB1 between patients with breast cancer and matched control subjects	Associations did not reach genome-wide significance (*P* < 5e−8), for HLA-DQA1 (imputed six 2-digits HLA alleles) and HLA-DQB1 (imputed five 2-digits HLA alleles)

The studies applied a case–control design and used blood samples

^a^Results on the association of HLA alleles and breast cancer risk, using logistic regression, adjusted for age and the first 15 principal components (Supplementary Table 4)

### Subgroup analysis on the associations between HLA alleles and breast cancer risk

Similar results were observed when different subtypes of breast cancer were studied (ER-positive, ER-negative, HER2-positive, HER2-negative, early-stage, and late-stage) (Figs. 2B–E and 3; Supplementary Figs. 5B-E and 6; and Supplementary Table 5). Effect sizes (OR, adjusted for age and the first 15 PCs) observed ranged from 0.75 to 1.47, where 95% of the effect sizes observed were between 0.85 and 1.18 (Supplementary Table 5). The association between HLA-B*35:03 and early-stage breast cancer showed the largest effect size after additional adjustments for study and breast cancer risk factors (OR = 0.60, *P* = 0.040) (Supplementary Table 6).

## Discussion

It was suggested that the MHC region is associated with breast cancer susceptibility studies of European populations [5, 13]. In contrast to the European genome-wide association study, we observed no significant association (*P* < 5e−8) between variants in the MHC region (imputed 175 classic class I and II HLA alleles) and breast cancer risk in our Asian study of 12,901 breast cancer cases and 12,583 controls (smallest *P* = 1.08e−3, HLA-C*12:03) [5].

Gourley et al. [14] reported that while studies showed no consistent association between HLA class I type (HLA-A, HLA-B and HLA-C) and breast cancer risk, certain HLA class I types may be more common in specific disease subclasses. Zhao et al. [15] reported down-regulation of HLA class I genes (which comprise HLA-A, HLA-B, or HLA-C) on CD4(+) and on CD8(+) T lymphocytes in breast cancer patients when as compared with healthy controls. However, we did not observe any significant association between any of the inferred HLA class I alleles and breast cancer risk, nor with any of the breast cancer subtypes in our Asian study population. Compared to other cancers, MHC class I mutations are less associated with the expression of killer lymphocyte effector genes in breast cancer [13]. Hence, possible differences in expression may be missed when studying HLA alleles (not expression).

Our systematic review identified one Asian study examining the relationship between HLA class I alleles and breast cancer risk [16]. Leong et al. [17] identified HLA-A*31 (6.8% in cases and 0% in controls; Fisher's exact test, *P* = 0.020) as a risk allele for breast cancer in 59 invasive ductal carcinoma breast cancer patients and 77 controls without breast cancer in Malaysia. In a subset of Malaysian women in our study, HLA-A*31 was not associated with breast cancer risk (adjusted OR = 0.77, *P* = 0.166; data not shown). It should be noted that the Malaysia study determined HLA-A expression using the Biotest HLA-A Sequence-Specific primers (SSP), while only imputed HLA alleles were interrogated in our study [17].

While it is conceivable that HLA genes play critical roles in immune surveillance against breast cancers, there are many other studies that show no consistent link between HLA and breast cancer [18]. Results from our study are in agreement with the two Asian studies on HLA class II alleles in the systematic review which reported the lack of associations between HLA alleles and breast cancer risk [16, 19]. Contrary to this, alleles from MHC class II sub-region were found to be associated with breast cancer risk in studies on populations of European ancestry (HLA-DQB1*03 [20], HLA-DRB1*10:01 [21] HLA-DRB1*11:01 [21], and HLA-DRB1*13 [20]) and Middle-Eastern populations (HLA-DQB1*02 [14, 22, 23], HLA-DRB1*03 [22], HLA-DQB1*06 [22], HLA-DRB1*07 [14], HLA-DRB1*12 [24], HLA-DRB1*13 [22], and HLA-DRB1*18:01 [25]) populations. It should be noted that previously published results have small sample sizes (largest sample size of 216 cases and 216 controls), which makes validation of the results challenging [16, 17, 19]. It is noteworthy that HLA alleles are exceedingly diverse, and may be confined to certain ethnic groups and geographical locations [26]. Indeed, we observed that by accounting for study populations, significant associations were attenuated and no longer significant, suggesting that correction for population stratification is essential in the testing of the associations.

This is the largest study to date to examine the associations between the MHC region and breast cancer risk in Asians (12,901 breast cancer cases and 12,583 controls). However, there are several limitations worth noting. Validation of the imputed HLA loci was not performed by the direct typing of HLA loci. An average error rate of between three to six percent has been reported for the prediction of high-resolution (four-digit) HLA alleles using imputation software [27]. Due to weaker linkage disequilibrium between rare variants, the imputation of rare HLA alleles is challenging. Variants with MAF > 1% in were filtered out prior to HLA imputation, and only imputed HLA alleles with MAF > 1% were retained in our analyses, which resulted in the exclusion of many informative alleles. In addition, non-classical HLA alleles were not studied, as the imputation software used imputes only classical HLA alleles (class I and class II). The accuracy of HLA imputation is dependent on the reference panel used [12]. The ethnic representativeness of the reference panel used for HLA loci imputation may limit the generalizability of our findings. While the chosen Pan-Asian reference panel is large, it may not represent the ethnic groups of our study population fully [27]. As there were no genome-wide significant results, we did not perform orthogonal experiments to verify the findings.

In conclusion, imputed class I and II HLA alleles were not associated with breast cancer risk in our Asian sample. Direct measurement of HLA gene expressions may be required to further explore the associations between HLA genes and breast cancer risk.

## Supplementary Information

Below is the link to the electronic supplementary material.

Supplementary file1 (XLSX 3996 kb)

Supplementary file2 (DOCX 15 kb)

## Data Availability

The genetic and clinical data used in this study were obtained via a data request (concept #686) to the Breast Cancer Association Consortium (BCAC). All data requests can be directed to the BCAC data access committee (http://bcac.ccge.medschl.cam.ac.uk/bcacdata/).
