# Supplementary material for: Relevance of the MHC region for breast cancer susceptibility in Asians
Source: Breast Cancer. 2022 May 11;29(5):869–79. doi: 10.1007/s12282-022-01366-w (PMC9385763; doi:10.1007/s12282-022-01366-w)
Supplement: Supplementary file 2 — Supplementary file2 (DOCX 15 kb) [file 12282_2022_1366_MOESM2_ESM.docx]

**Supplementary methods
*A systematic review of studies on HLA in Asian breast cancer patients***

The aim of this review is to identify HLA alleles (classic class I and II types) that are associated with the risk of developing breast cancer in humans. We conducted a systematic review on 7 July 2021 using PubMed. Full-text written in English (where available) was considered for all 14 articles identified using the keywords searched in **sTable 1**. We excluded eleven articles: three articles that were not in English, one without full-text, one protocol paper, one reported on cell lines, two studies were not on the risk of developing breast cancer, and three studies were not about the classes of HLA alleles of interest (**sTable 2**).

**sTable 1.** Keywords used in the systematic review done on 7 July 2021, using PubMed.

|  | **Keywords** | **Number of articles** |
| --- | --- | --- |
| #1 | ((HLA[Title/Abstract]) OR (Human Leukocyte Antigens[MeSH Terms])) AND (cancer[Title/Abstract]) | 7,391 |
| #2 | (((((((((((((((asia[Title/Abstract]) OR (asia*[Title/Abstract])) OR (Singapore[Title/Abstract])) OR (Malaysia[Title/Abstract])) OR (Thailand[Title/Abstract])) OR (Japan[Title/Abstract])) OR (China[Title/Abstract])) OR (India[Title/Abstract])) OR (India*[Title/Abstract])) OR (Japan*[Title/Abstract])) OR (Chinese*[Title/Abstract])) OR (Thai*[Title/Abstract])) OR (Malay*[Title/Abstract])) OR (Singapore*[Title/Abstract])) OR (Taiwan[Title/Abstract])) OR (Taiwan*[Title/Abstract])) OR (Hong Kong[Title/Abstract]) | 1,018,578 |
| #1 AND #2 | ((((HLA[Title/Abstract]) OR (Human Leukocyte Antigens[MeSH Terms])) AND (cancer[Title/Abstract])) AND (breast[Title])) AND (((((((((((((((((asia[Title/Abstract]) OR (asia*[Title/Abstract])) OR (Singapore[Title/Abstract])) OR (Malaysia[Title/Abstract])) OR (Thailand[Title/Abstract])) OR (Japan[Title/Abstract])) OR (China[Title/Abstract])) OR (India[Title/Abstract])) OR (India*[Title/Abstract])) OR (Japan*[Title/Abstract])) OR (Chinese*[Title/Abstract])) OR (Thai*[Title/Abstract])) OR (Malay*[Title/Abstract])) OR (Singapore*[Title/Abstract])) OR (Taiwan[Title/Abstract])) OR (Taiwan*[Title/Abstract])) OR (Hong Kong[Title/Abstract])) | 14 |

**sTable 2. Reasons for excluding articles.**

| **Title** | **Reason for exclusion** | **Citation** |
| --- | --- | --- |
| [Relationship of breast cancer and HLA in Japanese females] | Not in English | Gan No Rinsho. 1990 Jan;36(1):29-33. |
| [Immunohistochemical study on HLA expression of human breast cancer] | Not in English | Nihon Geka Gakkai Zasshi. 1990 Apr;91(4):508-14. |
| [Association of HLA with breast cancer and its ER status] | Not in English | Zhonghua Zhong Liu Za Zhi. 1989 Jan;11(1):19-21. |
| HLA alleles in pre-menopausal breast cancer patients from western India | No full text | Indian J Med Res. 2006 Sep;124(3):305-12. |
| Relationship between HLA-DRB1 gene polymorphism and breast cancer: A protocol for systematic review and meta-analysis | Protocol | Medicine (Baltimore). 2021 Mar 26;100(12):e25078. doi: 10.1097/MD.0000000000025078. |
| Identification of HLA-A24-restricted CD8(+) cytotoxic T-cell epitopes derived from mammaglobin-A, a human breast cancer-associated antigen | Cell lines | Hum Immunol. 2012 Jan;73(1):11-6. doi: 10.1016/j.humimm.2011.10.017. Epub 2011 Oct 23. |
| Association of HLA-G 3'UTR 14-bp Ins/Del polymorphism with breast cancer among South Indian women | Not in the classes of HLA of interest | J Clin Pathol. 2020 Aug;73(8):456-462. doi: 10.1136/jclinpath-2019-205772. Epub 2019 Dec 3. |
| The most important questions in cancer research and clinical oncology : Question 1. Could the vertical transmission of human papilloma virus (HPV) infection account for the cause, characteristics, and epidemiology of HPV-positive oropharyngeal carcinoma, non-smoking East Asian female lung adenocarcinoma, and/or East Asian triple-negative breast carcinoma? | Not in the classes of HLA of interest | Chin J Cancer. 2017 Jan 16;36(1):13. doi: 10.1186/s40880-016-0168-1. |
| Relationship between traditional Chinese medicine constitutional types with chemotherapy-induced nausea and vomiting in patients with breast cancer: an observational study | Not in the classes of HLA of interest | BMC Complement Altern Med. 2016 Nov 9;16(1):451. doi: 10.1186/s12906-016-1415-3. |
| Comparison of tumor-infiltrating lymphocytes between primary and metastatic tumors in breast cancer patients | Not studying breast cancer risk | Cancer Sci. 2016 Dec;107(12):1730-1735. doi: 10.1111/cas.13101. |
| Human leukocyte antigen as a predictive factor of developing bilateral breast cancer in Japanese women | Not studying breast cancer risk | Breast J. 2006 Sep-Oct;12(5 Suppl 2):S201-3. doi: 10.1111/j.1075-122X.2006.00335.x. |
